# On the gEUD biological optimization objective for organs at risk in Photon Optimizer of Eclipse treatment planning system

**DOI:** 10.1002/acm2.12224

**Published:** 2017-11-20

**Authors:** Antonella Fogliata, Stephen Thompson, Antonella Stravato, Stefano Tomatis, Marta Scorsetti, Luca Cozzi

**Affiliations:** ^1^ Radiotherapy and Radiosurgery Department Humanitas Research Hospital and Cancer Center Milan Rozzano Italy; ^2^ Varian Medical Systems Oncology Systems Palo Alto CA USA; ^3^ Biomedicine Faculty Humanitas University Milan Rozzano Italy

**Keywords:** biological optimization, gEUD, IMRT, optimization objective, VMAT

## Abstract

Inverse planning optimization using biologically based objectives is becoming part of the intensity modulated optimization process. The performances and efficacy of the biologically based gEUD (generalized Equivalent Uniform Dose) objective implemented in the Photon Optimizer (PO) of Varian Eclipse treatment planning system have been here analyzed. gEUD is associated with the parameter *a* that accounts for the seriality of a structure, being higher for more serial organs. The PO was used to optimize volumetric modulated arc therapy (VMAT) plans on a virtual homogeneous cylindrical phantom presenting a target and an organ at risk (OAR). The OAR was placed at 4 mm, 1 and 2 cm distance, or cropped at 0, 2 and 4 mm from the target. Homogeneous target dose of 60 Gy in 20 fractions was requested with physical dose‐volume objectives, while OAR dose was minimized with the upper gEUD objective. The gEUD specific *a* parameter was varied from 0.1 to 40 to assess its impact to OAR sparing and target coverage. Actual head and neck and prostate cases, with one parotid and the rectum as test OAR, were also analyzed to translate the results in the more complex clinical environment. Increasing the *a* parameter value in the gEUD objective, the optimization achieved lower volumes of the OAR which received the highest dose levels. The maximum dose in the OAR was minimized well with *a* values up to 20, while further increase of *a* to 40 did not further improve the result. The OAR mean dose was reduced for the OAR located at 1 and 2 cm distance from the target, enforced with increasing *a*. For cropped OARs, a mean dose reduction was achieved for *a* values up to 3–5, but mean dose increased for higher *a* values. The optimal choice of the parameter *a* depends on the mutual OAR and target position, and seriality of the organ. Today no significant compendium of clinical and biological specific *a* and gEUD values are available for a wide range of OARs.

## 1 INTRODUCTION

Intensity modulation radiotherapy planning, in both fixed beam setting (IMRT) and volumetric modulated arc setting (VMAT), uses inverse optimization processes generally based on physical dose or dose‐volume (DV) parameters. However, the planning criteria based on physical dose (or DV) constraints are a crude surrogate of any biological index that would better reflect the clinical goals. This makes the physical DV surrogates possibly inadequate to describe the radiation response of the tissues, and suboptimal to obtain a dose distribution that would aim to reflect more biological objectives.^1^, ^2^

Dose‐response models could be mechanistic, attempting to mathematically describe biological processes of cell survival, or phenomenological, empirically fitting available data. In the first group there are the normal tissue complication probability (NTCP) based model. The Lyman‐Kutcher‐Burman model^3^ described the dose‐response curve with three parameters, based on uniform irradiation; in this case, dose volume histogram (DVH) reduction algorithms were necessary to account for the non‐uniform dose distribution (an overview of those reduction models is, e.g., in^4^). Differently, the Relative Seriality model^5^ described the radiation response according to the damage and recovery of organ functional subunits. But the models which are the most used in the currently available biologically‐based treatment planning belong to the phenomenological approach. In particular, the Equivalent Uniform Dose (EUD) concept proposed by Niemierko,^6^ and its extension to gEUD (generalized EUD),^7^ provides a single metric for reporting non‐uniform dose distribution, using gEUD as a single organ specific parameter to account for the biological response according to the delivered dose distribution in that organ.

The inclusion of biologically‐based constraints in the optimizer cost function driving the inverse planning process, inherently incorporating a specific volume effect, could allow shaping the dose distribution by balancing the amount of volume receiving different dose levels. However, the result of an inverse planning optimization depends on the complex interplay of all the terms of the cost function. It is essential in the clinical practice to understand the effects of the biologically based objectives in controlling the dose distribution, and to know which is the desirable final dose distribution for a proper use of the underlying model.

The introduction of biologically based treatment planning, or a combination of physical and biological DV criteria in inverse planning objectives has been explored by the AAPM Task Group 166,^1^ describing the commercially available solutions at the time of publication.

A recent tool has been introduced in the new Photon Optimizer (PO), the inverse planning engine for both IMRT and VMAT implemented in the Eclipse™ planning system (Varian Medical Systems, Palo Alto, CA, USA) since its version 13.5, not described yet in the AAPM report. This tool makes available the gEUD objective as well as a variety of most general DV objectives in the same optimization process.

Aim of the present work is to investigate the results, from a clinical perspective, of the performance and efficacy of the biologically based gEUD objective in Eclipse™ in the inverse planning process, as it is implemented in the current optimization engine. Experiments were conducted for a simplified phantom with the aim to achieve some specific dose sparing inside given organs. The same was applied to clinical cases of head and neck (with a parotid as organ at risk, OAR) and prostate (with the rectum as OAR) to check and compare the consistency with real clinical application.

## 2 MATERIALS AND METHODS

### 2.A The gEUD, generalized Equivalent Uniform Dose

The gEUD is defined as:^6^, ^7^

$$gEUD={\left(\sum_{i}v_{i}D_{i}^{a}\right)}^{1/a}$$

where *v*_*i*_ is the fractional organ volume receiving a dose *D*_*i*_ and *a* is a parameter that describes the volume effect. For a→−∞ (negative *a* values, down to −40 is available in practice), gEUD approaches the minimum dose, and can be used for tumors. For a=1, gEUD equals the mean dose, and could be used in place of the mean objective for parallel organs. For a→+∞ (high *a* values, up to 40 in practice), gEUD tends to the maximum dose, and could be used to drive high (maximum) dose of serial organs.

The parameter *a* is organ specific, yet there are few specific studies to estimate the parameter values for the most important critical structures. However, it is related to the parameter *n* describing the volume effect in the Lyman‐Kutcher‐Burman NTCP model, as n=1/a. This last parameter has been widely analyzed, and a summary overview can be found in Luxton et al.^8^

**Figure 1:**
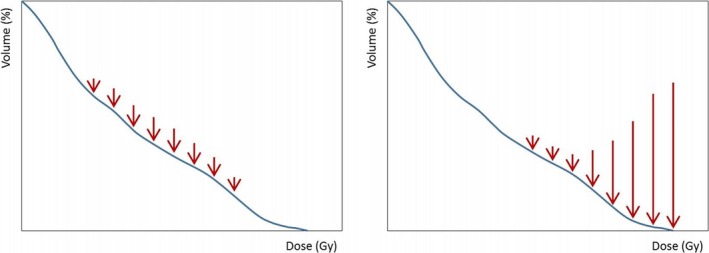
The gEUD optimization objective concept. The red arrows indicate the strengths applied to DVH for different *a* values of gEUD objective: low (*a* = 1) in the left panel, and high (*a* > 10) values in the right panel.

The concept of the gEUD optimization during inverse planning optimization is depicted in Fig. 1: for the DVH on the left, the *a* parameter is equal to 1 and the optimization force is directed to reduce the volume receiving mid‐dose levels, while, on the right DVH of the figure, the gEUD optimization with a high *a* parameter is shown to force the decrease in the structure volume receiving the higher dose levels. Regarding the specific implementation of the minimization of a convex optimization function (or non‐convex of a global objective function), it is part of the non‐disclosed implementation of the whole optimization engine.

### 2.B The optimization objectives in PO

A number of different optimization objectives are available in PO (here used in its version 13.6). There are the physical constraints as Upper, Lower, and Mean objectives used, respectively, to: limit the dose level in a defined portion of the structure volume, define a minimum dose level that a certain target volume should receive, define the mean dose that should not be exceeded for the structure.

The biological objectives are: Upper gEUD, Lower gEUD, and Target gEUD. The parameter *a* can vary from +0.1 to +40 for Upper gEUD, from −40 to +1, excluding 0, for Lower gEUD and Target gEUD.

The exploration of the lower and target gEUD objectives is out of the scope of the present work, which aims to evaluate the capability of this tool to modulate, with one single objective, the shape of an OAR DVH. The target dose homogeneity was optimized in the current work with the only use of a lower and an upper physical DV constraint.

In the cost function, the different objectives are similarly handled in the Eclipse™ optimization engine as follows:

$$cost\left(D\right)=W\times {\left(D_{\mathrm{actual}}-D_{\mathrm{expected}}\right)}^{2}$$

$$cost\left(\mathrm{Mean}\right)=W\times {\left(D_{{\mathrm{mean}}_{\mathrm{actual}}}-D_{{\mathrm{mean}}_{\mathrm{expected}}}\right)}^{2}$$

$$cost\left(gEUD\left(a\right)\right)=W\times {\left(gEUD{\left(a\right)}_{\mathrm{actual}}-gEUD{\left(a\right)}_{\mathrm{expected}}\right)}^{2}$$

where *W* is a normalized non‐linear function of priority, the *actual* values refer to the parameter during the optimization, the *expected* values refer to the settled objective. The total cost function is the summation of all the *cost* terms selected for optimization.

### 2.C The phantom study design

**Figure 2:**
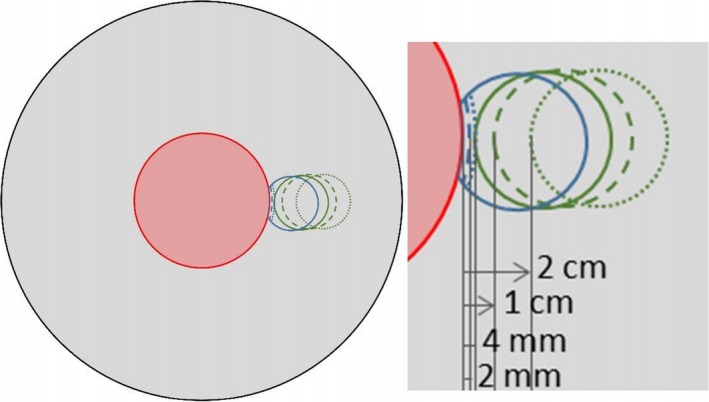
Virtual phantom layout: axial view and zoom onto the OARs.

A virtual phantom with homogeneous Hounsfield Unit (HU) assignment equal to 0 was generated in Eclipse. It had cylindrical shape of 30 cm diameter and 50 cm long. In the middle of the phantom, a cylindrical target was delineated, of 10 cm diameter and length. As organs at risk (OAR), different cylinders (4 cm diameter and 5 cm long) were delineated on the left of the target: with target to OAR centres distance of 6.5 cm, cropped at the target (OAR_0mmCrop), 2 mm from the target (OAR_2mmCrop), 4 mm from the target (OAR_4mmCrop), and 4 mm, 1 and 2 cm distant from the target (OAR + 4mm, OAR + 1cm, OAR + 2cm), as shown in Fig. 2. The OAR_4mmCrop and OAR + 4mm have the same distance from the target, but only in one point, since OAR_4mmCrop has a larger part of surface that has been cropped to 4 mm from the target: some differences are hence expected in the results, being, geometrically speaking, the OAR + 4mm easier to spare than OAR_4mmCrop. No OAR overlapped to the target was delineated, since this would have compromised the target coverage in the overlapping region, mixing the simple gEUD use with more complex trade‐offs that would require at least a different balance of the priority values.

Simple VMAT plans (RapidArc) with one single full arc of 6 MV from a Varian TrueBeam linear accelerator equipped with Millennium 120‐MLC were optimized to deliver 60 Gy in 20 fractions to the target, with the following constraints:

1. Target: Lower objective volume = 100%, dose = 60 Gy, priority 100
2. Target: Upper objective volume = 0%, dose = 60 Gy, priority 100
3. OAR: Upper gEUD objective dose 15 Gy, priority 70, variable *a* = 0.1, 1, 2, 3, 5, 10, 20, 40.
4. Normal Tissue Objective: automatic, priority 100. This tool evaluates mean dose at concentric distances from targets and applies a penalty to voxels which exceed the mean dose of all voxels at the same distance. This generates a dose fall‐off outside the target with a rather constant gradient from the target surface.

An additional plan was generated with a Mean objective to the OAR (dose 15 Gy, priority 70), to compare with the plan with upper gEUD with *a* = 1. Although the gEUD(1) equals the mean dose, the possible differences between the two applications are here explored.

No interactions were allowed during the optimization, which was left running for all the four multiple resolution levels without modifications nor level hold. The optimization resolution was set to 2.5 mm (normal setting). Final dose calculations used Acuros dose calculation algorithm (version 13.6) with 2.5 mm grid size. The ascription of 0 HU, with no specific material assignment, corresponds to the human tissue composed by 3% of adipose and 97% of muscle skeletal tissue.^9^

Results on OAR dose distribution were reported as mean dose, gEUD (according to the varying *a* parameter), maximum point and near‐to‐maximum doses (as D_1%_, D_2%_, i.e., the dose received by 2% of the structure volume), D_20%_, D_50%_, V_15 Gy_ (volume receiving no more than 15 Gy dose level).

### 2.D The clinical cases

Three head and neck and three prostate patients were selected from the institutional database. A VMAT plan according to the internal rules was generated for each case: 4 arcs for head and neck to deliver two dose levels of 54.45, and 69.96 Gy in 33 fractions; 2 arcs for the prostate including seminal vescicles to deliver the hypofractionated treatment of 38 Gy in 4 fractions. Plans were normalized, also for full consistency, to mean target dose. One parotid and the rectum were chosen as the test OAR for gEUD optimization. New structures were generated: parotid and rectum cropped to the target (with no margin), and the same cropped 4 mm by the target. New plans were generated optimizing the test OAR with the mean objective, or the upper gEUD objective, varying the *a* parameter value (1, 2, 3, 5, and 10). The priority was not made changing, as well as all the other optimization objectives. During the optimization no interactions were allowed, while it was possible to hold each of the resolution levels to permit the optimizer to achieve a flat cost, as during the routine clinical procedure.

The same parameters analyzed in the phantom study were here evaluated, and compared with those.

## 3 RESULTS

### 3.A Phantom study

**Figure 3:**
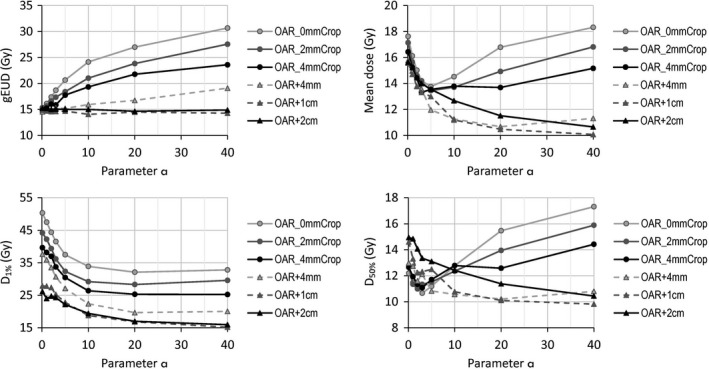
Dosimetric parameters as a function of the *a* value. Upper line: gEUD and mean dose; lower line near‐to‐maximum dose D_1%_ and D_50%_.

**Figure 4:**
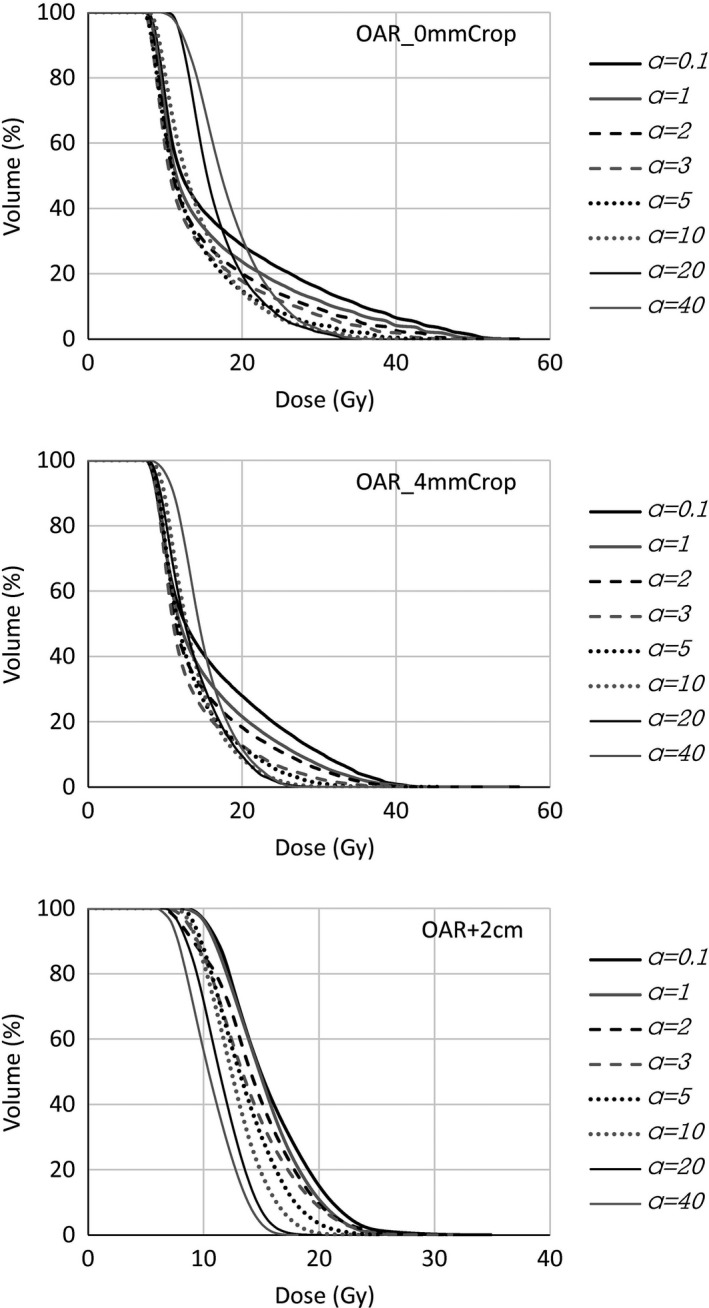
DVH for OAR_0mmCrop, OAR_4mmCrop, and OAR + 2cm from optimizations with different *a* values.

In Fig. 3, some dosimetric results are shown for the various OAR at different distances from the target, as a function of the parameter *a* applied to the upper gEUD objective during the plan optimization varied from 0.1 to 40. Figure 4 reports the DVHs of OAR for the three cases of OAR_0mmCrop, OAR_4mmCrop, and OAR + 2cm, i.e., the structures for which the optimization was driven.

The requested value of 15 Gy for the gEUD was achieved for any value of the parameter *a* only when the OAR was located far from the target (2 cm distance in the exercise), where a homogeneous dose was requested with higher priority. In the cases where the OAR was located closer to the target, the requested gEUD value was achieved only for very small *a* value, while increasing with *a*. The mean dose (and similarly D_50%_) to the same OAR structure decreased for all OAR‐target distances, until an *a* value of 3–5; then, for *a* 10 and higher, the mean structure dose increases with *a* for OAR distances cropped 0 to 4 mm from the target, while for OAR positioned from 4 mm to 2 cm from the target, the mean dose continuously decreases. On the other hand, the behavior of the maximum and near‐to‐maximum doses is different and continuously decreases with *a*. However, the maximum dose reaches an approximate plateau (around an *a* value of 10–20), beyond which the optimizer is not able to significantly reduce the dose further.

The trade‐off due to the increased OAR sparing for high doses achieved by increasing the *a* value, especially when the OAR structure is close to the target, could be twofold. On one side, there is a diminished target homogeneity and coverage, as highlighted in Table 1 with the Standard Deviation and V_95%_ parameter (volume receiving at least 95% of the prescription dose) for the target structure. This is evident for the OAR_0mmCrop, but also for the OAR_4mmCrop, while for the OAR + 2cm, the target coverage is not affected by the increased *a* value. On the other side, the dose spillage (EI, external volume index), defined as the non‐target volume receiving the prescription dose divided by the volume of the target, has no clear trend relative to the *a* parameter value, apart from showing the lowest dose spillage for *a* = 0.1, and constant EI for distant OARs.

**Table 1:** Target results as a function of *a*. Trg SD is the standard deviation (in Gy) inside the target, Trg V_95%_ is the percentage of the target volume receiving at least 95% of the prescription dose. EI is the external volume index to estimate the dose spillage outside the target

*a*	OAR_0mmCrop			OAR_4mmCrop			OAR + 2cm		
	Trg SD	Trg V_95%_	EI	Trg SD	Trg V_95%_	EI	Trg SD	Trg V_95%_	EI
0.1	1.078	99.1%	1.5%	1.042	99.2%	1.6%	1.038	99.3%	1.3%
1	1.177	98.7%	1.9%	1.049	99.2%	2.3%	1.026	99.3%	1.3%
2	1.249	98.7%	2.2%	1.105	99.0%	1.8%	1.075	99.1%	1.3%
3	1.376	98.1%	1.4%	1.095	99.1%	2.1%	1.003	99.5%	1.4%
5	1.575	97.4%	1.8%	1.278	98.6%	1.8%	0.998	99.4%	1.3%
10	1.907	96.3%	2.5%	1.568	97.3%	3.2%	1.051	99.1%	1.4%
20	2.194	95.9%	2.3%	1.614	97.3%	3.1%	1.024	99.2%	1.3%
40	2.530	92.8%	1.4%	1.925	96.3%	3.5%	0.972	99.5%	1.4%

Regarding DVHs in Fig. 4, the cases of OARs cropped to the target (0 to 4 mm) present a smooth decreasing behavior for *a* values up to 3. From *a* = 3 on, the gEUD cost function compromises the low doses in the attempt of reducing the high dose levels. Ofnote is the substantial increase in the dose received by the majority of the OAR volume in the *a* = 40 case, even though the maximum dose (maximum point dose or D_1%_) was not further reduced with respect to the *a* = 20 case. Conversely, for a distant structure, for the same dose level requested, all the DVH smoothly decrease for *a* from 0.1 to its maximum value 40.

In the geometries studied, where an OAR is cropped to relatively distant, there is not much to gain from using an a parameter higher than 10 or 20. When using an *a* of 40, the maximal or near maximum dose of the OAR is nearly the same as that for a = 10 or a = 20, yet the mean dose is higher and target coverage is worse.

Comparing the plans optimized using the upper gEUD objective with *a* = 1 vs. the mean dose objective, differences were clearly present, showing that the different objectives (gEUD vs. mean dose) led to different terms in the cost function. Results are summarized in Table 2. The variations were not negligible for the OAR maximum dose (or even near‐to‐maximum doses), where the mean objective resulted in higher maximum doses for OAR positioned 1 or 2 cm from the target, although the mean OAR dose was within 1.5% in all analyzed cases. Also the target dose homogeneity (evaluated as the standard deviation parameter), was shown to be better for upper gEUD objective plans in the cases where the OAR was cropped from the target.

**Table 2:** Comparison between OAR dosimetric parameter using mean or gEUD(*a* = 1) objectives

	OAR D_1%_ [Gy]		OAR D_max_ [Gy]		OAR D_mean_ [Gy]		Target SD [Gy]	
	gEUD(1)	Mean	gEUD(1)	Mean	gEUD(1)	Mean	gEUD(1)	Mean
OAR_0mmCrop	47.49	47.60	54.46	53.15	16.12	16.16	1.177	1.219
OAR_2mmCrop	42.21	42.09	48.37	47.99	15.65	15.81	1.141	1.179
OAR_4mmCrop	38.16	37.49	47.28	44.92	15.25	15.06	1.049	1.071
OAR + 4mm	35.89	34.58	44.30	43.33	14.97	14.89	1.073	1.044
OAR + 1cm	27.76	29.00	31.50	33.60	14.70	14.90	0.970	0.992
OAR + 2cm	23.97	24.94	27.75	31.89	15.22	15.06	1.026	1.023

### 3.B Clinical cases

**Figure 5:**
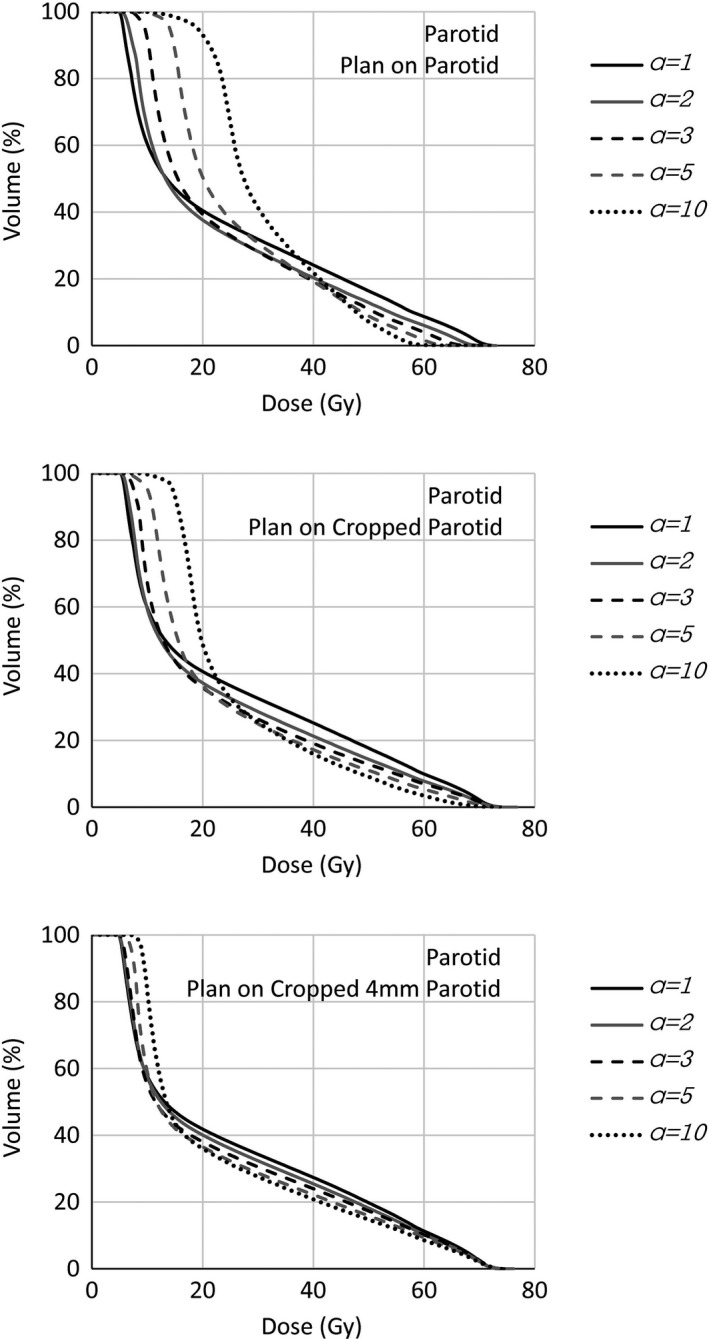
Whole parotid DVHs of head and neck case for different gEUD *a* value settings. Plans optimized on the whole parotid, OAR cropped by the target, OAR cropped 4 mm by the target.

The dose distributions and DVHs of the clinical cases confirmed the general message reported for the phantom case. In Fig. 5, the DVHs of the whole parotid (the clinically interesting structure) of one of the head and neck patients are shown, for different *a* values, in the cases where the optimization was based on the parotid cropped to the target, cropped 4 mm by the target, as well as the whole parotid (16% of the volume overlapped the target) as is often used in clinical practice.

For increasing *a* values, the DVHs of the clinical case present the same trend as the corresponding cases in the phantom exercise, decreasing the volume receiving high doses at the price of the volume receiving low doses. This similar behavior showed the applicability of the above simple phantom results to more complex situations. The 16% of the parotid volume receiving high dose values (the prescription dose levels were 54.45 and 69.96 Gy) is, with good probability, the volume overlapping with the target, and hence, for a good coverage, should receive high doses. This is accomplished only in the case where the plans were optimized on the parotid cropped 4 mm from the target, whichever the *a* value (in particular, the mean parotid doses were, in those cases, 25.1, 23.3, 23.9 Gy for *a* = 1, 5, 10, respectively). Opposed is the case of optimization on the whole parotid. At increasing *a*, as expected, the optimizer would spare more the high doses, not able then to spare at low dose levels; in this case the target coverage is more and more compromised and the mean parotid dose increases for higher *a* values (e.g., 23.9, 26.5, 31.3 Gy for *a* = 1, 5, 10, respectively). The other analyzed cases showed similar results.

**Figure 6:**
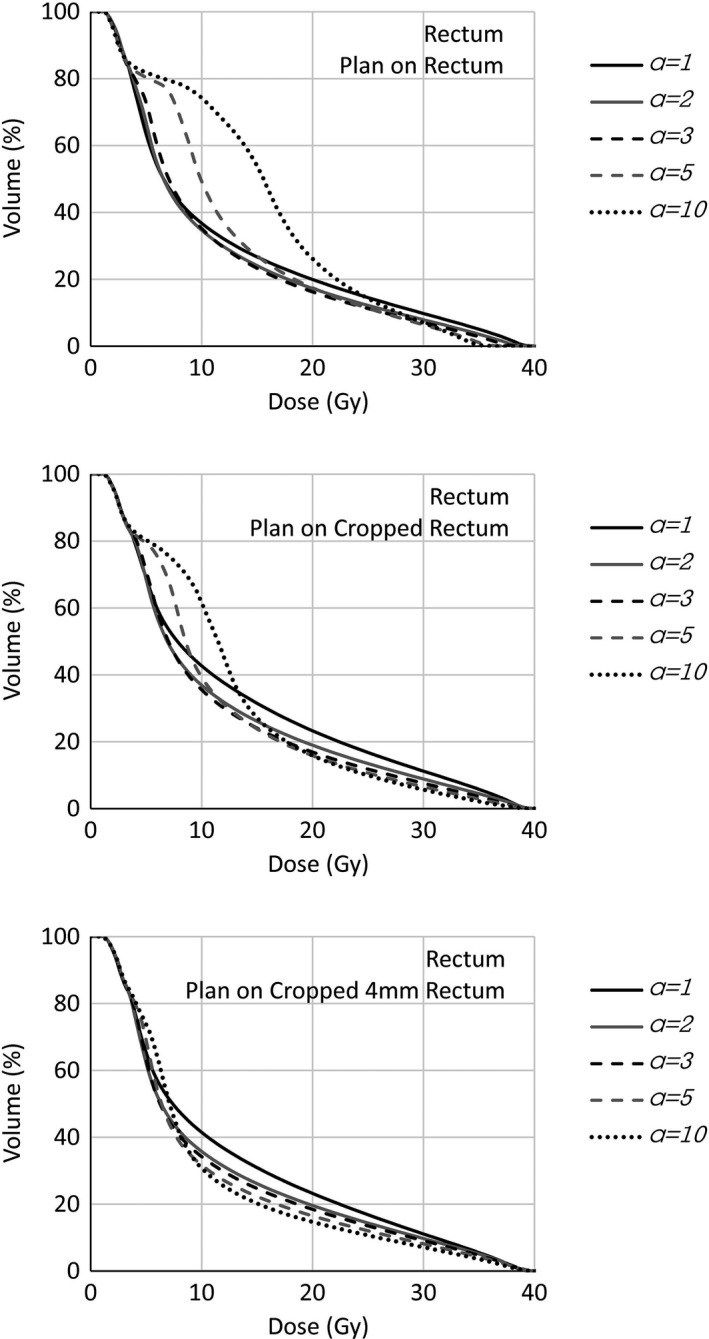
Whole rectum DVHs of prostate case for different gEUD *a* value settings. Plans optimized on the whole rectum, OAR cropped by the target, OAR cropped 4 mm by the target.

**Figure 7:**
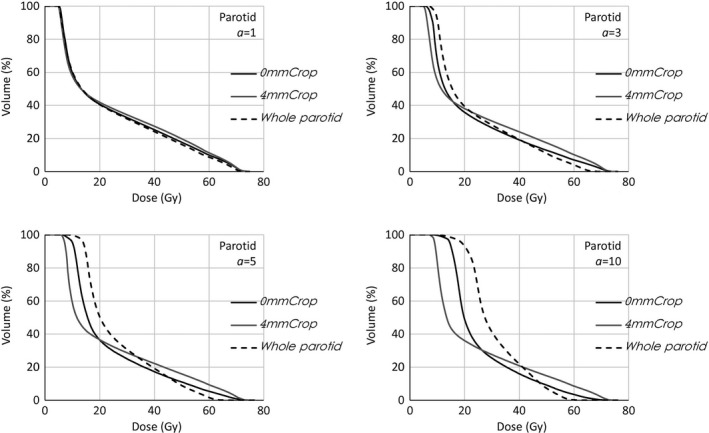
Whole parotid DVHs optimized on different OARs (whole OAR, cropped by the target, cropped 4 mm by the target). *a* gEUD parameter set to 1, 3, 5, 10.

**Figure 8:**
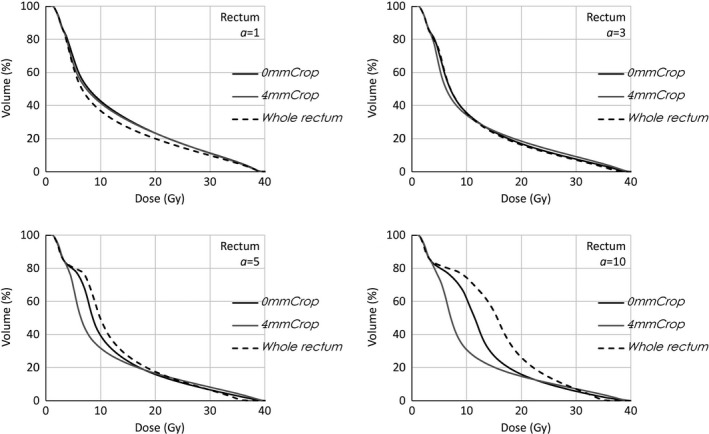
Whole rectum DVHs optimized on different OARs (whole OAR, cropped by the target, cropped 4 mm by the target). *a* gEUD parameter set to 1, 3, 5, 10.

In Figs. 6 and 7 present the corresponding of Figs. 5 and 8, for the rectal DVHs of one of the prostate cases are shown (for this patient, the rectum overlapping the target was 10% of the rectal volume). Very similar description is applied to those graphs. Ofnote, in this case, the visual inspection of isodose distribution, presented much clearer the overlapping region receiving some target underdose. This could suggest to the possibility to use the entire OAR during the optimization process with a gEUD optimization objective with rather high *a* value if the planning strategy on the overlapping volume is to compromise the target coverage.

## 4 DISCUSSION

The use of the biological optimization parameter called “upper gEUD” was evaluated for OAR structures at different distances from the target during VMAT optimization in the Eclipse™ PO optimizer. It shows to be a powerful objective tool to improve the OAR sparing without compromising the target coverage and homogeneity when applied with an *a* value selected according to the structure seriality and target/OAR geometry.

Biological DV objectives have been explored for other planning systems or ad‐hoc optimization engines, presenting similar results. The first applications of the gEUD concept^10^, ^11^ showed that the EUD‐based optimization was capable of improving the sparing of the critical structure, keeping the same target coverage, although for target dose homogeneity care should be paid for possible hot spots that has to be managed with additional constraints, balancing the trade‐offs between target homogeneity and OAR sparing.^11^ Some clinical application of the use of gEUD‐based optimization have been published. Widesott et al.^12^ optimized prostate and head and neck IMRT plans, obtaining promising results using the parameter *a* = 1 in all cases, while not varying the *a* value to shape the optimized DVH.

Mihailidis et al.^13^ optimized IMRT plans using gEUD‐based objectives for OARs comparing the results to plans optimized using physical DV constraints for breast and chest wall planning treatments. The authors concluded that gEUD‐based plans allow greater sparing of the OARs while maintaining equivalent target coverage, with a reduction, e.g., from 22% to 18% of the V_20 Gy_ parameter for the ipsilateral lung. In 2012, Dogan^14^ compared head and neck VMAT plans using Pinnacle with physical DV constraints and gEUD‐based plans. The latter approach yielded 55% reduction in near‐to‐maximum cord dose, and 35% reduction in mean parotid dose. By varying the *a* value, they reported an increase of MU for gEUD‐based plans by 12%, 19%, 21% for *a* set to 1, 5, and 10, respectively. In our current study, no net trend with *a* was shown for MU. Although a general modest increase with *a* seems to be present, this was not systematic. Again on Pinnacle, Lee^15^ evaluated on 10 IMRT patients the differences between physical and gEUD‐based optimization for bilateral breast planning, reporting a better OAR sparing with biological features, using fixed *a* values of 1 and 3 for lung and heart, respectively (Pinnacle default values for those structures). On Eclipse, but using the specific biological optimization module based in TCP and NTCP (both Lyman and serial models), Kan^16^ reported that for nasopharyngeal cancer IMRT planning, the biological optimization yielded comparable TCP, relative to physical optimization, while presenting more hot spots in the target, better NTCP for parotids, no significant difference for serial organs. Similar conclusions were outlined by Feng^17^ for cervical cancer planning using IMRT and the same biological optimization module in Eclipse.

The biological optimization using gEUD, for both target and OAR, has been used by Cabrera G. et al.^18^ as the basis of their model formulation for beam angle and fluence map optimization of IMRT plans, taking advantage of the favorable optimization properties of the gEUD function, as convexity and positive homogeneity.

From the results reported in this work, the use of gEUD optimization objectives in the common PO optimizer (not the specific biological optimization module) could substantially help in improving the plan quality and OARs sparing. The simplified phantom geometry here analyzed allowed to visualize the possible OAR dose reduction (low or high dose levels) considering the specific *a* value adopted, and the distance (together with some geometrical differences in OAR cropping or simple distance between two cylinders) between OAR and target, separating the results from any specific anatomical or density inhomogeneity coming from any specific patient and site.

Two main aspects were underlined: on one side the specific anatomy influences the DVH shape; the force to apply to the high dose levels, translated into the choice of the *a* value, depends on the mutual position of the OAR and the target. On the other side, there is the understanding of the *a* value as a biological parameter. Some published values of the *n* parameter of the Lyman‐Kutcher‐Burman NTCP model could be considered as a starting value for the gEUD optimization as a “true” biological solution. However, there is a lack of knowledge in which is the gEUD(*a*) tolerance value for each specific organ. Since the gEUD(*a*) value depends on the DVH shape, there is no correlation between e.g., the mean dose and the gEUD. This makes more difficult a correct use of the gEUD objective, once applied the proper organ‐specific *a* value, as there is no published data on gEUD(*a*) tolerance levels for specific organs.

In this view, in light of more specifically biological evaluations, the gEUD objective in Eclipse™ can be safely used since it reduces the OAR dose to all involved dose levels for *a* values from 1 to ~5. For larger *a* values, attention should be paid case by case, since, depending on the structures geometry and mutual locations, the DVH could be lower for some dose range and higher for other ranges.

For a more biologically conscious use of the gEUD‐based optimization, and to give meaning to the gEUD dose in relation to a specific *a* parameter for each specific organ, we need clinical studies evaluating the patient toxicity related to gEUD and the *a* parameter for the most important critical structures. This will allow reducing better the doses to OAR, in the low or high dose range, where clinically and biologically it is more relevant in the specific structure, thanks to an improved knowledge of the biological and clinical effect of the radiation. For the moment, for the specific use of *a*, we could start from the fact that *a = 1/n*, and *n* values have been widely published and summarized.^8^

The simplicity of the proposed phantom exercise allowed the understanding of the performance of the sole gEUD upper objective, without mixing or confounding different effects deriving from other sources anatomy related. However, the use of this tool to specific clinical cases was confirming the trends read in the phantom setting, now able to possibly distinguish between the tool performance and the anatomical specific features.

## 5 CONCLUSIONS

The gEUD optimization objective implemented in the Eclipse PO optimizer has shown to be a powerful instrument to spare the OARs without reducing the target coverage. A better understanding of the correlation between the *a* parameter and the OAR radiobiology remains advisable.

## CONFLICT OF INTEREST

Stephen Thompson is an employee of Varian Medical Systems, and there is no significant financial support for this work that could have influenced its outcome. Luca Cozzi acts as a Scientific Advisor to Varian Medical Systems and is Clinical Research Scientist at Humanitas Cancer Center.
